# Organ-Sparing Surgical Management of a Uriniferous Perinephric Pseudocyst Associated with a Calyceal Diverticulum in a Cat with a Solitary Kidney

**DOI:** 10.3390/vetsci13020154

**Published:** 2026-02-04

**Authors:** Seung-Joon Lee, Ji-Hyun Park, Hyeong-Jun Yim, Earl Choi, Geon-Ung Byun, Chang-Hwan Moon, Dongbin Lee

**Affiliations:** 1 Institute of Animal Medicine, College of Veterinary Medicine, Gyeongsang National University, Jinju 52828, Republic of Korea; dr203@naver.com (S.-J.L.); 24qkr@naver.com (J.-H.P.); byun4510@naver.com (G.-U.B.); ch.moon@gnu.ac.kr (C.-H.M.); 2 24-Hour Changwon Referral Veterinary Hospital, Changwon 51388, Republic of Korea; gudwns1340@naver.com (H.-J.Y.); earl.choi.dvm@gmail.com (E.C.)

**Keywords:** urinary leakage, computed tomography urography, omental plug technique, solitary kidney

## Abstract

Uriniferous perinephric pseudocysts (PNPs) are a rare condition in cats, primarily managed by nephrectomy to eliminate persistent urinary leakage. Organ preservation is critical in cases with solitary kidneys. This report describes a cat with congenital absence of the right kidney that developed a uriniferous PNP secondary to abnormal communication between the calyceal diverticulum and subcapsular space. A 6-year-and-11-month-old neutered male Ragdoll cat presented with abdominal distension and lethargy. Ultrasonography revealed an extensive subcapsular perinephric fluid and a cystic lesion adjacent to the renal pelvis. Contrast-enhanced computed tomography with excretory urography directly demonstrated the time-dependent passage of contrast medium from the renal pelvis into the calyceal diverticulum and subsequent leakage into the subcapsular space, allowing precise identification of the renal leakage pathway. Based on these findings, an operation was performed using a non-vascularized free omental plug inserted into the diverticular opening and secured using capsular sutures. Postoperative drainage resolved rapidly, renal function normalized, and no recurrence was detected during long-term follow-up of up to 465 days. To the best of our knowledge, this is the first report to describe an organ-sparing surgical approach that directly addresses the renal leakage pathway in feline uriniferous PNPs.

## Simple Summary

Perinephric pseudocysts are fluid-filled spaces that develop around the kidneys. In rare cases, these cysts contain urine due to urinary leakage, and nephrectomy is typically required to prevent ongoing extravasation. However, this approach is not feasible in cats with a single kidney. We report the case of a congenitally single-kidney cat that developed a urine-filled perinephric pseudocyst secondary to abnormal leakage from a small renal outpouching. Advanced computed tomography imaging clearly demonstrated urinary leakage. Rather than performing a nephrectomy, the defect was sealed using an omental patch. The fluid resolved quickly, renal function recovered, and no recurrence was observed. This case demonstrates that careful imaging and kidney-preserving surgery can be successful alternatives for selected cases.

## 1. Introduction

A perinephric pseudocyst (PNP) is characterized by the accumulation of fluid within a fibrous sac surrounding one or both kidneys, with the fluid most commonly located in the subcapsular or extracapsular spaces [1,2,3,4]. Due to these morphological characteristics, the term “pseudocyst” is used, as the lesion lacks an epithelial lining [1,2,4]. PNPs are classified into four categories—uriniferous, lymphatic, hemorrhagic, and transudative—based on the nature of the cystic fluid [1,2]. Analysis of the cystic fluid, including creatinine concentration, total protein concentration, and nucleated cell count, is indicated to aid in differentiation [1,2]. Uriniferous PNPs arise from urinary leakage into the perirenal space and are exceedingly rare in cats, with only sporadic cases reported [1,2,4]. The most common clinical signs in cats with PNPs include gradually progressive abdominal distension and a palpable abdominal mass [1,2]. Moreover, nonspecific clinical signs such as anorexia, weight loss, vomiting, polyuria, and polydipsia are frequently observed [1,2,3,4].

A calyceal diverticulum is a cystic structure that communicates with the renal collecting system through a narrow neck [5]. The condition is most often identified as an incidental finding and is usually asymptomatic [5,6]. However, owing to its communication with the collecting system, a calyceal diverticulum may potentially serve as a source of urinary stasis and structural complications [5,6,7]. In veterinary sciences, calyceal diverticula can be challenging to differentiate from simple cystic lesions, and their clinical significance as well as associated complications remain insufficiently documented [5].

High-resolution imaging modalities, including contrast-enhanced computed tomography (CT), are essential for distinguishing renal cysts, calyceal diverticula, and perinephric fluid accumulations [4,5]. These techniques enable the evaluation of lesion attenuation and assessment of communication with the collecting system [5]. In cases of uriniferous PNPs in particular, CT excretory urography is superior to conventional excretory urography for identifying and localizing the underlying lesion responsible for urinary leakage [4,5].

Surgical management of a PNP may vary depending on the lesion’s location, size, and relationship with adjacent anatomical structures [1,2]. In previously reported cases of uriniferous PNPs, nephrectomy was performed to remove lesions responsible for persistent urinary leakage [8,9,10]. This approach inherently sacrifices renal function. To the best of our knowledge, no previous reports have described an organ-sparing procedure that directly targets the anatomical source of urinary leakage while preserving renal function.

This case report describes an extremely rare presentation of a uriniferous PNP resulting from abnormal communication between a calyceal diverticulum and the subcapsular space in a cat with a congenital solitary kidney, and details its successful surgical management using an organ-sparing surgical approach, defined as renal-sparing focal therapy without resection of renal parenchyma.

## 2. Case Presentation

A 6-year-and-11-month-old, 9.2 kg, client-owned neutered male Ragdoll cat with a body condition score of approximately 7/9 was referred for evaluation of abdominal distension and lethargy. Abdominal ultrasonography performed at the referring hospital revealed a large volume of fluid surrounding the left kidney and congenital absence of the right kidney.

Upon presentation, the cat was lethargic, and vital signs were unremarkable. Physical examination revealed a marked enlargement of the left kidney and mild abdominal discomfort on palpation.

Serum biochemical analysis revealed an increased serum creatinine concentration of 3.5 mg/dL (reference range, 0.8–2.4 mg/dL). Mild hyperglycemia was also noted, with a blood glucose concentration of 179 mg/dL (reference range, 71–159 mg/dL), accompanied by a slight increase in globulin concentration to 5.5 g/dL (reference range, 2.8–5.1 g/dL). No other clinically relevant abnormalities were identified on blood examination.

**Figure 1:**
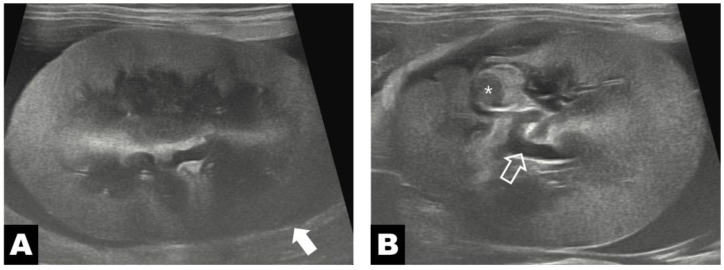
Preoperative ultrasonography of the left kidney. (**A**) Perinephric fluid accumulation within the pseudocyst wall (white arrow) around the kidney. (**B**) The diverticulum (asterisk) appears as cystic cavities near the renal pelvis (white hollow arrow).

Abdominal ultrasonography, performed using a diagnostic ultrasound system (ARIETTA 70; Hitachi-Aloka, Tokyo, Japan) equipped with a high-frequency linear transducer (2–12 MHz), revealed extensive subcapsular fluid surrounding the entire left kidney between the renal parenchyma and capsule, with thinning of the renal cortex due to compressive effects (Figure 1A). As the cat had a solitary kidney, intrarenal regional measurements were compared. The preoperative renal volume was 34.08 cm^3^. At regions distant from the diverticular lesion, cortical and medullary thicknesses were 7.3 mm and 12.2 mm, respectively, with a medulla-to-cortex ratio of 1.67. In contrast, measurements obtained adjacent to the diverticular lesion demonstrated marked parenchymal thinning, with cortical thickness of 3.3 mm and medullary thickness of 6.9 mm, corresponding to an increased medulla-to-cortex ratio of 2.09. In addition, a narrow anechoic channel connecting the renal pelvis to the adjacent subcapsular space was identified, suggesting the presence of an open tract rather than an isolated closed cystic lesion (Figure 1B).

**Figure 2:**
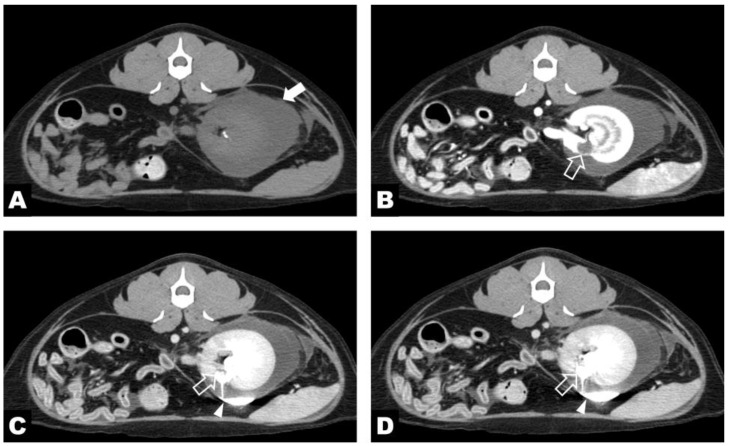
Computed tomography excretory urography images of the left kidney. Pre-contrast (**A**), post-contrast nephrogenic phase (**B**), and excretory phase images obtained at 5 (**C**) and 10 min (**D**) after contrast administration at the same cross-sectional level. On the pre-contrast image (**A**), an irregularly marginated subcapsular cavity adjacent to the renal pelvis is observed (white arrow). All images at the identical cross-sectional level demonstrate time-dependent opacification of the calyceal diverticulum and the associated cystic cavity (white hollow arrow, (**B**–**D**)). During the excretory phases (**C**,**D**), contrast medium passes from the renal pelvis into the calyceal diverticulum and progressively fills the subcapsular cavity (white arrowhead).

Contrast-enhanced CT and excretory urography were performed under general anesthesia using a 160-slice multidetector CT scanner (Aquilion Lightning 160; Canon Medical Systems, Tochigi, Japan). Anesthetic induction was achieved with propofol (2 mg/kg IV; Provive^®^ Inj., Myungmoon Pharm. Co., Seoul, Republic of Korea), and anesthesia was maintained with isoflurane (Ifran^®^; Hana Pharm. Co., Seoul, Republic of Korea). For contrast enhancement, a non-ionic iodinated contrast agent, iohexol (300 mg iodine/mL; Omnipaque^®^, GE Healthcare, Cork, Ireland), was administered intravenously at a dose of 600 mg iodine/kg using a power injector (2 mL/s). Peri-procedural therapy was administered at a maintenance rate using Plaju-OP (Plaju OP^®^ Inj., JW-pharma, Dangjin, Republic of Korea) to support renal perfusion during and after contrast administration. Contrast-enhanced CT and excretory urography demonstrated marked enlargement of the left kidney with extensive low-attenuation fluid surrounding the kidney within the subcapsular space (Figure 2A). A calyceal diverticulum, measuring approximately 9 mm in diameter, was identified on the ventromedial surface of the kidney (Figure 2B). Five minutes after contrast administration, the renal pelvis was opacified, followed by passage of contrast into the calyceal diverticulum via the collecting system. Progressive leakage of contrast into the subcapsular space through the open tract of the diverticulum was observed (Figure 2). The right kidney and associated renal vasculature were entirely absent, consistent with congenital renal agenesis.

Ultrasound-guided percutaneous aspiration of cystic fluid was also performed. The cat was positioned in lateral recumbency. Ultrasonographic guidance was performed by scanning the left mid-abdominal region in a transverse plane. A 23-gauge needle attached to a 5 mL syringe was advanced in-plane, parallel to the transducer, allowing continuous visualization of the needle tip during advancement. The needle was carefully guided into the fluid-filled space, and fluid was aspirated under real-time ultrasonographic monitoring. The aspirated fluid was yellowish-orange, with a total protein concentration of 27 g/L and a creatinine concentration exceeding 13.6 mg/dL, yielding a fluid-to-serum creatinine ratio greater than 3.9. The specific gravity was 1.010, and the total nucleated cell count was 790 cells/µL, consistent with urinous fluid. No bacterial growth was detected on either aerobic or anaerobic cultures of the cystic fluid.

Based on the imaging findings obtained on the day of admission, together with the results of additional diagnostic tests, the cat was diagnosed with a uriniferous perinephric pseudocyst secondary to persistent urinary leakage resulting from abnormal communication between the calyceal diverticulum and the subcapsular space. Given the cat’s solitary kidney, a renal-sparing focal surgical approach using omentalization was selected instead of nephrectomy, the conventional treatment for uriniferous PNPs. Supportive therapy, including maintenance-rate Plaju OP (Plaju OP^®^ Inj., JW-phrama, Dangjin, Republic of Korea) administration, was continued until surgery, which was performed approximately 4 h after the CT examination.

Surgery was performed under general anesthesia. Anesthetic induction was achieved with midazolam (0.15 mg/kg IV; Bukwang Midazolam Inj, Bukwang Pharm, Seoul, Republic of Korea) and butorphanol (0.15 mg/kg IV; Butophan Inj., Myungmoon Pharm, Seoul, Republic of Korea), followed by administration of propofol (6 mg/kg IV; Provive^®^ Inj., Myungmoon Pharm. Co., Seoul, Republic of Korea). The cat was then endotracheally intubated with a 4.5 mm endotracheal tube, and anesthesia was maintained with isoflurane (Ifran^®^; Hana Pharm. Co., Seoul, Republic of Korea). Cefotaxime (30 mg/kg IV; Unitaxime^®^ Inj., Union Pharm, Wonju, Republic of Korea) was administered as a preoperative antibiotic, and standard adjunctive medications, including atropine (0.02 mg/kg IM; Daehan Pharm, Seoul, Republic of Korea), were available if required. During anesthesia, continuous anesthetic monitoring showed that the heart rate was maintained between 120 and 140 beats/min, and non-invasive blood pressure was maintained between 70 and 80 mmHg, with no clinically significant cardiovascular or respiratory abnormalities observed throughout the procedure.

**Figure 3:**
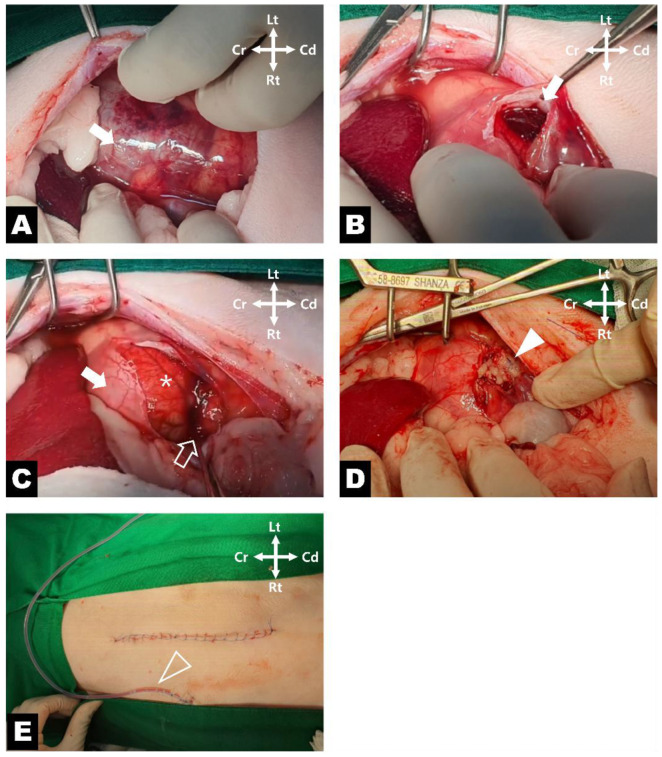
Intraoperative views during surgical management of the left kidney. (**A**) The left kidney is demonstrated surrounded by a markedly distended renal capsule (white arrow). (**B**) Appearance after aspiration of the accumulated fluid following capsulotomy (white arrow). (**C**) The renal capsule is incised to expose the renal parenchyma (white asterisk), and a communicating site between the capsule (white arrow) and the calyceal diverticulum is identified along the renal parenchyma (white hollow arrow). (**D**) The communicating tract is covered with non-vascularized free omental tissue and secured using capsular suturing (white arrowhead). (**E**) Placement of a closed suction drain exiting through a separate stab incision adjacent to the abdominal surgical site (white hollow arrowhead).

Surgery was performed via a ventral midline celiotomy. Following celiotomy, the left kidney was markedly enlarged, with a renal capsule that was thin, translucent, pale, extremely fragile, and prone to rupture (Figure 3A). Careful incision of the capsule using a No. 11 blade allowed evacuation of the accumulated fluid using suction, which precluded accurate measurement of the total volume. Following lavage, a widely dissected space between the renal capsule and parenchyma was identified (Figure 3B). Following capsulotomy, a calyceal diverticulum was identified on the medial surface, with its opening directly communicating with a defect in the renal capsule (Figure 3C). The opening of the diverticulum was left unsutured. To cover the opening of the diverticulum, a free omental graft matching the size of the defect was harvested. The distal end of the prepared omental graft was gently inserted into the diverticular opening in a plug-like fashion. The incised renal capsule was then repositioned over the omentum and secured with three mattress sutures using 6–0 polydioxanone (Figure 3D). Histopathological examination was not performed due to the high risk of renal parenchymal injury in a cat with a solitary kidney and the need to preserve the renal capsule intact for capsular closure as part of the surgical procedure [4,11]. A closed suction drain (Barovac^®^, SS200L, 8 Fr, Sewoon Medical, Seoul, Republic of Korea) was placed, and routine abdominal closure was performed.

Postoperative analgesia was provided using local infiltration of 0.5% bupivacaine (1 mg/kg SC; Myungmoon Bupivacaine^®^, Myungmoon Pharm, Seoul, Republic of Korea) along the incision line immediately after surgery, combined with tramadol (2 mg/kg, IV; Tramadol HCl Inj., Shinpoong Pharm, Seoul, Republic of Korea). Postoperative antimicrobial therapy consisted of cefotaxime (30 mg/kg IV; Unitaxime^®^ Inj., Union Pharm, Wonju, Republic of Korea) for 5 days. In addition, famotidine (0.5 mg/kg IV; Gaster^®^ inj., DongA-st, Seoul, Republic of Korea) was administered as a gastrointestinal protectant for 5 days. Intravenous fluid therapy was provided using Plaju OP (Plaju OP^®^ Inj., JW-phrama, Dangjin, Republic of Korea) at a maintenance rate during hospitalization.

On postoperative day 1, the drainage volume was relatively high (3.75 mL/kg/h). Thereafter, drainage gradually decreased, reaching 0.16 mL/kg/h by postoperative day 5 and the closed suction drain was maintained until postoperative day 6, when drainage had nearly completely resolved and the drain was removed. The cat’s overall activity and appetite gradually improved throughout hospitalization. On postoperative day 1, urine output, as measured through an indwelling urinary catheter, was maintained at 1.1 mL/kg/h, which was considered within the normal range. Based on the progressive decrease in drainage volume and concurrent clinical improvement, the postoperative drainage was considered an expected postoperative finding associated with intraoperative abdominal lavage performed during the surgical procedure. On serial blood tests, the serum creatinine concentration was 2.3 mg/dL immediately after surgery, progressively declining to 1.3 mg/dL on postoperative day 2 and 1.1 mg/dL on postoperative day 3, stabilizing within the reference range. During hospitalization, serum sodium concentrations remained relatively stable at 153–159 mmol/L (reference range: 150–162 mmol/L); meanwhile, serum chloride concentrations ranged from 115 to 121 mmol/L (reference range: 116–126 mmol/L). The serum potassium concentration was transiently reduced to 3.1 mmol/L (reference range: 3.5–4.8 mmol/L) on the day following surgery, indicating mild hypokalemia; however, it subsequently increased to 3.4 mmol/L on follow-up testing, and no clinically significant complications were observed. After this time point, renal function was not further assessed using laboratory parameters.

**Figure 4:**
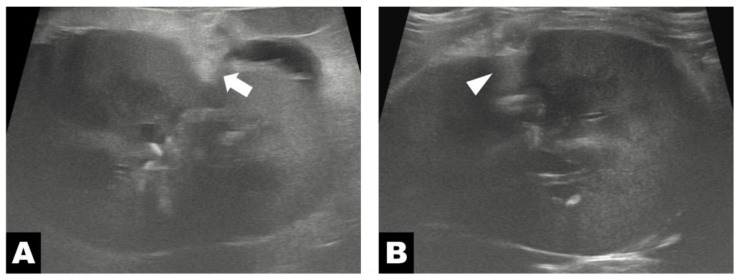
Postoperative ultrasonographic images of the left kidney. (**A**) On postoperative day 4, omental tissue (white arrow) is identified at the level of the renal parenchyma, with surrounding increased fat echogenicity. (**B**) Postoperative day 80, omental tissue (white arrowhead) is observed again at the same location, with no perinephric fluid accumulation observed.

Abdominal ultrasonography performed on postoperative day 4 demonstrated the presence of omental tissue adjacent to the renal parenchyma, along with increased echogenicity of the surrounding perinephric fat and a small amount of residual perinephric fluid (Figure 4A). At this time point, the renal volume was 29.33 cm^3^. Ultrasonographic measurements obtained at the region adjacent to the diverticular lesion revealed a cortical thickness of 4.7 mm and a medullary thickness of 6.0 mm, resulting in a medulla-to-cortex ratio of 1.28. On follow-up ultrasonography performed on postoperative day 80, the omental tissue remained visible at the same anatomical location (Figure 4B), whereas the previously noted increase in perinephric fat echogenicity and perinephric fluid accumulation had resolved. Follow-up evaluation showed a renal volume of 30.84 cm^3^, with cortical and medullary thicknesses of 4.5 mm and 6.5 mm, respectively, corresponding to a medulla-to-cortex ratio of 1.44, measured at the same diverticular-adjacent region. Long-term follow-up at 465 days postoperatively was based on owner-reported information obtained by telephone, during which no recurrence of clinical signs was noted.

## 3. Discussion

This case report describes a PNP associated with the formation of an open tract directly connecting the calyceal diverticulum to the subcapsular space in a cat with a solitary kidney. Using contrast-enhanced CT, the origin and progression pathways of the urinary leakage were identified, and the condition was successfully managed with an organ-sparing approach using omentalization.

The treatment options for PNPs include percutaneous fluid drainage, capsulectomy, omentalization, and nephrectomy [1,2,4,12,13]. However, in previously reported cases involving uriniferous pseudocysts, surgical management has largely focused on eliminating the presumed source of persistent urinary leakage rather than directly addressing the pseudocyst, with nephrectomy being the most commonly employed treatment approach [8,9,10]. Nephrectomy was not considered in this case due to the presence of a solitary kidney, necessitating an organ-sparing approach, prioritizing preservation of renal function. In this case, omentalization was selected as an alternative surgical technique capable of directly occluding the urinary leakage pathway while preserving renal tissue.

Several omentalization techniques have been described, including wrapping, patch, and plug methods using the omentum [12,13,14,15]. In previously reported feline PNP cases, omentalization has most commonly been performed using a vascularized pedicled omental flap, typically in the form of omental wrapping, with favorable outcomes [16]. In the present case, however, the lesion consisted of an open tract directly communicating between a calyceal diverticulum and the subcapsular renal space. Accordingly, the primary surgical objective was to achieve permanent closure through effective mechanical sealing of the leakage tract. Based on these lesion-specific characteristics and surgical goals, a free omental plug technique was selected as the primary surgical approach. The structural advantages of omental tissue in obliterating irregular spaces in various cavitary lesions have been repeatedly reported in the literature [14,15,16]. Owing to its thin and highly flexible nature, free omental tissue can be shaped three-dimensionally to conform to the defect, allowing dense packing of narrow and deep spaces and effective sealing of the cavity [14,15,16]. In addition, non-vascularized free omental tissue is gradually replaced by fibrous tissue over time; this technique is considered more suitable for promoting scar formation required for the long-term prevention of urinary leakage [17]. Although the non-vascularized free omental plug technique has not been previously reported in veterinary medicine, a similar concept has been described in human surgery. In that report, non-vascularized free omental tissue harvested during subtotal cholecystectomy was utilized as a plug in the gallbladder bed to prevent bile leakage [17]. This supports the concept that the omentum can function not only as a surface patch but also as an intracavitary packing material capable of effectively sealing high-risk leakage sites. The same principle was applied in the present case to pack the opening of the calyceal diverticulum, and no recurrence of urinary leakage was identified on ultrasonographic evaluation for up to 80 days postoperatively. Furthermore, key biological functions of omental tissues have been reported to be partially preserved, regardless of the vascularization status [16]. Recent experimental studies have demonstrated that non-vascularized free omental grafts significantly enhance healing strength in bone tissue models by promoting neovascularization, facilitating the rapid removal of tissue fluid and inflammatory byproducts, activating macrophages, and accelerating fibrosis and tissue remodeling [16]. Through these mechanisms, the free omental plug used in this case was expected to provide stable sealing of the leakage tract and promote an effective healing response, even within the narrow and deep subcapsular renal space. Nevertheless, the biological behavior of non-vascularized free omental grafts in the perinephric environment has not yet been fully elucidated, and further studies are required to clarify the underlying mechanisms.

PNPs reported in cats consist of non-inflammatory transudates that gradually accumulate within the subcapsular space [2,4]. In many cases, the underlying pathogenesis remains unclear [1,2,4]. In this case, the diagnosis of a uriniferous PNP was established using a combination of radiography, ultrasonography, cystic fluid analysis, and CT urography. Abdominal ultrasonography is a non-invasive and preferred modality for confirming the presence of a PNP [1,2,4]. Accumulation of anechoic fluid between the renal capsule and parenchyma is typically observed on renal ultrasonography and is considered virtually pathognomonic for a PNP [1,2,4]. Consistent with previous reports, these ultrasonographic features were also identified in this case. In evaluating the underlying cause of PNPs, particularly uriniferous PNPs, CT urography proves superior to conventional excretory urography for detecting and localizing the source of urinary leakage and is valuable for preoperative planning [4,5]. To the best of our knowledge, no previous studies have described the use of CT excretory urography in cats with solitary kidneys to demonstrate the direct leakage of urine from a calyceal diverticulum into the subcapsular space, resulting in PNP formation. In the current study, CT urography allowed precise identification of the opening site of the calyceal diverticulum, thereby minimizing unnecessary capsular incisions and facilitating a targeted application of the omental plug technique. Fluid analysis is an essential component of clinical management for differentiating the underlying causes of a PNP, particularly in suspected uriniferous cases [1,2,4]. A cystic fluid creatinine concentration higher than or at least twice the serum creatinine concentration is diagnostic of a uriniferous PNP [1,2,4]. In this case, the creatinine concentration of the cystic fluid far exceeded the diagnostic threshold, confirming a definitive classification as a uriniferous PNP.

Calyceal diverticula associated with contralateral renal agenesis have been reported in cats [5,7]. Embryologically, both conditions are believed to arise from failure of ureteric bud development [5,7]. Therefore, in this case, both the calyceal diverticulum and renal agenesis were likely of congenital origin. Such calyceal diverticula exhibit inherent anatomical fragility due to thinning of the renal parenchyma within the diverticulum [6]. Due to this vulnerability, cases of iatrogenic rupture of calyceal diverticula caused by increased intrapelvic pressure during ureteroscopic lithotripsy have been reported in human medicine [6].

The abnormal communication between the diverticulum and the subcapsular space observed in this case, along with the associated fluid leakage, can be interpreted as a consequence of this structural weakness. Consequently, this report illustrates how a calyceal diverticulum may manifest clinically. Accordingly, in veterinary patients with similar lesions, precise delineation of the anatomical structure using ultrasonography and CT, together with strategies aimed at minimizing pressure and mechanical stress during all diagnostic and therapeutic manipulations, is warranted.

## 4. Conclusions

This case represents the first report of a uriniferous PNP resulting from communication with a calyceal diverticulum in a cat with a solitary kidney, as well as the first successful organ-sparing surgical management of a feline uriniferous PNP. CT urography enables diagnostic decision making by directly demonstrating abnormal communication between the diverticulum and the subcapsular space, whereas the free omental plug technique effectively obliterates the complex three-dimensional cavity and achieves long-term prevention of urinary leakage. This case highlights the clinical utility of imaging-based diagnosis and non-vascularized omental plug techniques in veterinary patients with similar lesions, providing an important reference for establishing future diagnostic and therapeutic strategies for feline uriniferous PNPs.

## Data Availability

The original contributions presented in this study are included in the article. Further inquiries can be directed to the corresponding author.

## Abbreviations

The following abbreviations are used in this manuscript:

CT	Computed tomography
PNP	Perinephric pseudocyst
